# Development of Meloxicam-Human Serum Albumin Nanoparticles for Nose-to-Brain Delivery via Application of a Quality by Design Approach

**DOI:** 10.3390/pharmaceutics12020097

**Published:** 2020-01-25

**Authors:** Gábor Katona, György Tibor Balogh, Gergő Dargó, Róbert Gáspár, Árpád Márki, Eszter Ducza, Anita Sztojkov-Ivanov, Ferenc Tömösi, Gábor Kecskeméti, Tamás Janáky, Tamás Kiss, Rita Ambrus, Edina Pallagi, Piroska Szabó-Révész, Ildikó Csóka

**Affiliations:** 1 Faculty of Pharmacy, Institute of Pharmaceutical Technology and Regulatory Affairs, University of Szeged, H-6720 Szeged, Hungary; tamas.kiss@pharm.u-szeged.hu (T.K.); arita@pharm.u-szeged.hu (R.A.); edina.pallagi@pharm.u-szeged.hu (E.P.); revesz@pharm.u-szeged.hu (P.S.-R.); csoka@pharm.u-szeged.hu (I.C.); 2 Department of Chemical and Environmental Process Engineering, Budapest University of Technology and Economics, H-1111 Budapest, Hungary; balogh.gyorgy@pharm.u-szeged.hu (G.T.B.); gdargo@mail.bme.hu (G.D.); 3 Faculty of Pharmacy, Department of Pharmacodynamics and Biopharmacy, University of Szeged, H-6720 Szeged, Hungary; ducza@pharm.u-szeged.hu (E.D.); ivanov.anita@pharm.u-szeged.hu (A.S.-I.); 4 Faculty of Medicine, Department of Pharmacology and Pharmacotherapy, University of Szeged, H-6720 Szeged, Hungary; gaspar.robert@med.u-szeged.hu; 5 Faculty of Medicine, Department of Medical Physics and Informatics, University of Szeged, H-6720 Szeged, Hungary; marki.arpad@med.u-szeged.hu; 6 Interdisciplinary Excellence Centre, Department of Medical Chemistry, University of Szeged, H-6720 Szeged, Hungary; tomosi.ferenc@med.u-szeged.hu (F.T.); kecskemeti.gabor@med.u-szeged.hu (G.K.); janaky.tamas@med.u-szeged.hu (T.J.)

**Keywords:** meloxicam, HSA nanoparticles, nose-to-brain delivery, quality by design, physical stability, in vivo animal studies, rapid equilibrium dialysis, PAMPA

## Abstract

The aim of this study was to optimize the formulation of meloxicam (MEL)-containing human serum albumin (HSA) nanoparticles for nose-to-brain via a quality by design (QbD) approach. Liquid and dried formulations of nanoparticles containing Tween 80 and without the surfactant were investigated. Various properties, such as the Z-average, zeta potential, encapsulation efficacy (EE), conjugation of MEL and HSA, physical stability, in vitro dissolution, in vitro permeability, and in vivo plasma and brain distribution of MEL were characterized. From a stability point of view, a solid product (Mel-HSA-Tween) is recommended for further development since it met the desired critical parameters (176 ± 0.3 nm Z-average, 0.205 ± 0.01 PdI, −14.1 ± 0.7 mV zeta potential) after 6 months of storage. In vitro examination showed a significantly increased drug dissolution and permeability of MEL-containing nanoparticles, especially in the case of applying Tween 80. The in vivo studies confirmed both the trans-epithelial and axonal transport of nanoparticles, and a significantly higher cerebral concentration of MEL was detected with nose-to-brain delivery, in comparison with intravenous or per os administration. These results indicate intranasal the administration of optimized MEL-containing HSA formulations as a potentially applicable “value-added” product for the treatment of neuroinflammation.

## 1. Introduction

In recent years, several studies have pointed out that neuroinflammation plays a pivotal role in the progression of neuropathological changes observed in neurodegenerative diseases like Alzheimer’s disease [1]. Thus, anti-inflammatory drugs, especially non-steroid anti-inflammatory agents (NSAIDs), can be administered with the therapeutic target to treat neuroinflammation, thereby delaying the progression of the disease [2,3]. NSAIDs protect the mitochondria, and therefore hold significant promise against Alzheimer’s disease. They depolarize the mitochondria and inhibit calcium uptake, even at low concentrations, due to the ionizable carboxylic group, which is similar to the mild mitochondrial uncouplers [4].

The main challenge of neurodegenerative disease therapy is to pass the drug through the blood–brain barrier (BBB). Since the oral administration of NSAIDs results in poor brain penetration, it is favorable to use alternative methods of drug delivery. Nasal delivery provides a promising administration route for the following reasons: the nasal mucosa has a large surface area that is available for drug absorption and the drug can be absorbed directly into the brain and therefore avoids the efflux mechanism of BBB, which offers the possibility of local treatment of the neuroinflammation [5,6]. Furthermore, the easy accessibility of the nasal cavity allows for quick self-administration, thus further improving patient expectations. Nasal absorption can occur through the following pathways [7,8,9]. In the respiratory region, drugs can either enter the systemic circulation or be directly transported to the brain tissues through the trigeminal nerve [10]. In the olfactory region, drugs can be transported or diffuse directly to the brain through the olfactory mucosa, which is considered to be the most important direct pathway [11]. Some aspects must be taken into consideration when formulating for intranasal (IN) administration. The pH of the compositions should be similar to that of the nasal human mucosa (5.0 to 6.5) in order to avoid irritation; tonicity should be high enough to promote absorption, but not excessively so to avoid toxicity to the nasal epithelium or enhancement of mucociliary clearance. Applying viscosity enhancers increases the contact time with the nasal mucosa but might also decrease the drug diffusion [12]. Furthermore, the volume also has limitations, only up to 200 μL can be administered IN [13].

Experimental studies have shown that meloxicam (MEL), a selective COX-2 inhibitor enolcarboxamide derivative with anti-inflammatory, analgesic, and antipyretic effects, has potential therapeutic effects on improving the anti-amnesic activity through inhibiting lipid peroxidation, increasing the effect of endogenous antioxidant enzymes [14] and decreasing acetylcholinesterase activity in the brain [15]. Compared to other earlier NSAIDs, MEL causes less irritation to the gastrointestinal tract and has an appropriate safety profile [16].

Albumin nanoparticles are able to improve drug absorption through the nasal mucosa. Human serum albumin (HSA) is a versatile, biodegradable transport peptide for targeted drug delivery [17,18]. As a colloidal drug carrier, it can bind small molecules and peptide drugs through its charged amino acids and carboxyl and amino groups. The mechanisms of binding can be physical or covalent binding of the drug to albumin through a ligand or protein-binding group, fusion of the drug with albumin, and encapsulation of drugs into albumin nanoparticles [19]. The therapeutic effect of albumin nanoparticles was researched initially in tumor therapy. They can penetrate the tumors because of an albumin transport pathway mediated by the glycoprotein gp60 located on the endothelial cell surface of tumors. Albumin transport pathways, such as those mediated by gp60, are found on the surface of endothelial cells in peripheral capillaries [20]. However, brain endothelial cells have relatively low expression, and the BBB prevents albumin from crossing. In order to bypass the BBB, recent studies show evidence for the uptake of albumin across the nasal epithelium. Uptake occurred through a saturable transport system to all regions of the brain rapidly, with the highest levels detected in the striatum and the olfactory bulb [21]. The novelty of the present study is the optimization of an organic solvent and cytotoxic crosslinker-free formulation of an innovative MEL containing HSA nanoparticles by applying a quality by design (QbD) approach with improved brain targetability compared to intravenous (IV) administration. Our research group has already described formerly successful nose-to-brain applications of MEL via nanoparticles, such as an IN spray [22] and gelling solid dispersion [23]. *R*-flurbiprofen (another NSAID) containing HSA nanoparticles has already shown promising results in nose-to-brain drug delivery [24,25].

The QbD concept is a risk-and-knowledge-based quality management method used generally in the pharmaceutical industry as QbD elements are now regulatory requirements of the submissions [26]. The QbD method focuses on the profound preliminary design of the target product and the practical experiments are based on the results of the risk assessments (RAs) [27]. When the QbD is applied in the development, the first step is the prior definition of the quality target product profile (QTPP), which contains the essential parameters from the point of view of the patient and the clinical setting. It is a prospective summary of the quality characteristics of the product that will ideally be achieved. The next step is the selection of the parameters that critically influence the QTPPs. These are the critical quality attributes (CQAs) related to the safety and efficacy of the product, namely the critical material attributes (CMAs) and the critical process parameters (CPPs) related to the selected production method. The key element of a QbD-guided development is the RA (it can be initial, recurrent/updated, or finalized) [28], which results in ranked CQAs and CPPs regarding their critical effect on the targeted product quality. The classical QbD model was successfully applied previously by the authors in several cases of pharmaceutical early developments [29,30,31,32], as well as in a nasal-formulation [33] and a peptide-containing product [34].

Our aim was to optimize the formulation of MEL-containing HSA nanoparticles for nose-to-brain delivery as a potentially applicable “value-added” product against neuroinflammation. The QTTP and its influencing parameters were determined using an RA. The formulation of nanoparticles was optimized using hydrodynamic diameter (Z-average) and encapsulation efficacy (EE) as the two quality-determining product parameter through a Box–Behnken experimental design. The effect of Tween 80 was studied in the formulation of MEL-containing HSA nanoparticles with different methods. The conjugation of MEL and HSA was investigated with FT-IR. In vitro dissolution studies with rapid equilibrium dialysis (RED) and in vitro parallel artificial membrane permeability assays (PAMPAs) were carried out. In vivo animal studies were performed to determine the blood–brain distribution and pharmacokinetic parameters of IN administered nanoparticles.

## 2. Materials and Methods

### 2.1. Materials

MEL (4-hydroxy-2-methyl-n-(5-methyl-2-thiazolyl)-2H-benzothiazine-3-carboxamide-1,1-dioxide) as a model drug was obtained from EGIS Ltd. (Budapest, Hungary). HSA (lyophilized powder, purity >97%), Tween 80 (Tween), phosphate buffered saline (PBS) powder, disodium hydrogen phosphate (Na_2_HPO_4_), sodium dihydrogen phosphate (Na_2_HPO_4_), l-α-phosphatidylcholine (PC), and cholesterol (CHO) were purchased from Sigma Aldrich Co. Ltd. (Budapest, Hungary). The analytical grade solvents acetonitrile (MeCN), dimethyl sulfoxide (DMSO), dodecane, formic acid, and trifluoroacetic acid (TFA) were purchased from Merck KGaA (Darmstadt, Germany). Piroxicam, the internal standard for the HPLC method, was purchased from the Alfa Aeasar Co. (Alfa Aeasar GmbH & Co. KG, Karlsruhe, Germany). In all experiments, distilled water was purified using the Millipore Milli-Q^®^ (Merck Ltd., Budapest, Hungary) 140 Gradient Water Purification System.

### 2.2. Initial Risk Assessment and Knowledge Space Development

The first step is the determination of the QTPP of the target product, which is the essential element to successfully completing the QbD-based initial RA. Table 1 shows the defined requirements, as QTPP and their elements, of the aimed albumin-containing product.

In the second step, the CQAs of the final product and the CPPs of the selected production method were identified. Simultaneously, a primary knowledge space development was made as part of the QbD methodology, which meant the collection, systematization, and visualization of all the product and production-relevant information. The LeanQbD^®^ software (QbD Works LLC, Fremont, CA, USA, www.qbdworks.com) was used for the RA procedure. In this procedure, the first part was the interdependence rating among the QTPPs and CQAs, as well as among the CQAs and CPPs. A three-level scale was used to describe the relationship between the parameters: “high” (H), “medium” (M), or “low” (L). Then, a risk occurrence rating of the CPPs (or probability rating step) was made, applying the same three-grade scale (H/M/L) ranking structure. As the output of the initial RA evaluation, Pareto diagrams were generated by the software presenting the numerical data and the ranking of the CQAs and CPPs according to their potential impact.

### 2.3. Optimization of the Preparation of MEL-HSA Nanoparticles

The preparation of MEL-HSA nanoparticles was optimized using a Box–Behnken experimental design. The key parameters were defined based on the RA. The selected independent variables, namely the amounts of MEL, HSA, and Tween 80, were considered as the critical parameters in the preparation process with an effect on the Z-average and EE. These three experimental factors were varied in the design, at 3 levels in 15 runs. The content of MEL ranged from 1 to 3 mg/mL, while the content of HSA ranged from 5 to 15 mg/mL and the amount of Tween 80 was set from 0 to 6 mg/mL. This design was employed to investigate the quadratic response surface and to construct a second-order polynomial model using TIBCO Statistica^®^ 13.4 (Statsoft Hungary, Budapest, Hungary). The relationship of the variables on the response could be analyzed using the following second-order equation:

(1) $$Y=\beta_{0}+\beta_{1}x_{1}+\beta_{2}x_{2}+\beta_{3}x_{3}+\beta_{12}x_{1}x_{2}+\beta_{13}x_{1}x_{3}+\beta_{23}x_{2}x_{3}+\beta_{11}x_{1}^{2}+\beta_{22}x_{2}^{2}+\beta_{33}x_{3}^{2},$$

where *Y* is the response variable; *β*_0_ is a constant; *β*_1_, *β*_2_, and *β*_3_ are linear coefficients; *β*_12_, *β*_13_, and *β*_23_ are interaction coefficients between the three factors; and *β*_11_, *β*_22_, and *β*_33_ are quadratic coefficients. The 3D response surface plot has a function of understanding the main effects and the interaction effects of two factors, maintaining all other factors at a fixed level. The 3D response surface plots for Z-average (*Y*_1_) and entrapment efficiency (*Y*_2_) were plotted according to the regression model by keeping one variable at the center level. The significance of the variables and interactions were evaluated using analysis of variance (ANOVA). Differences were considered significant when the *p*-value was less than 0.05.

### 2.4. Preparation of MEL-Albumin Nanoparticles

**Figure 1:**
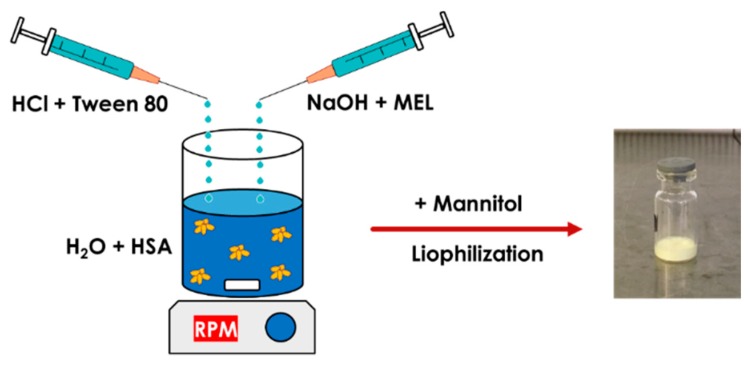
Preparation of meloxicam (MEL)-HSA nanoparticles using a modified coacervation method.

MEL-albumin nanoparticles were prepared using a modified coacervation method based on the pH-dependent coacervation of HSA (Figure 1) [38]. MEL has a week acidic character (p*K_a_* = 3.43 [39]); therefore, it is poorly water soluble, but highly soluble in an acidic or alkaline medium. Liquid formulations were prepared as follows: Tween-80 was dissolved in a 4 mL HCl solution (0.1 M), whereas MEL was dissolved in a 4 mL NaOH solution (0.1 M) and HSA was dissolved in 8 mL of purified water. Then, the MEL-NaOH solution was slowly added dropwise (2 mL/min) to the HSA solution under constant stirring with 800 rpm at 4 °C. Next, Tween 80-HCl solution was added dropwise to the MEL-HSA solution. After homogenization of the solution with a magnetic stirrer (800 rpm), additional HCl solution was added to adjust the pH to 5.6, and as a result, the formulation became turbid. Then, the formulation was kept under constant stirring at 4 °C for 12 h incubation to obtain a nanoparticle dispersion. Two types of formulations were prepared and further investigated: one without Tween (MEL-HSA) and one containing Tween (MEL-HSA-Tween). Solid preparations were prepared the same way as the liquids, with the difference that 0.80 g (5% *w*/*w*) of mannitol was added to the liquid formulations as a cryoprotectant, then the solution was transferred to 1.5 mL vials and freeze-dried at −25 °C for 12 h under a 0.013 mbar pressure and then kept at 25 °C for 3 h for secondary drying to obtain lyophilized powders using a Scanvac Coolsafe labor freeze-dryer (Labogene, Lynge, Denmark). Both the liquid and powder ampoules were stored at 5 ± 3 °C until further investigations.

### 2.5. Z-Average and Zeta Potential Determination

The Z-average, polydispersity index (PdI), zeta potential, and physical stability of MEL-albumin nanoparticles were determined separately before and after lyophilization using dynamic light scattering (DLS) in a folded capillary cell using a Zetasizer Nano ZS (Malvern Instruments, Malvern, UK) at 25 °C. The refractive index of the nanoparticles was 1.712. The lyophilized samples were reconstituted with 1.5 mL of purified water. All measurements were repeated three times, and the average values of each were used.

### 2.6. Determination of Encapsulation Efficacy

MEL-albumin nanoparticles were separated from the aqueous medium via centrifugation using a Hermle Z323K high performance refrigerated centrifuge (Hermle AG, Gossheim, Germany) at 15,000 rpm at 4 °C for 30 min. The amount of free MEL was determined in the clear supernatant, which was diluted 10-fold with purified water using an Agilent 1260 HPLC (Agilent Technologies, San Diego, CA, USA). For the stationary phase, a Kinetex^®^ C18 column (5 μm, 150 mm × 4.6 mm (Phenomenex, Torrance, CA, USA)) was used. The concentration of MEL was determined in the injected 10 µL samples at 30 °C. The mobile phase A was a 0.065 M aqueous KH_2_PO_4_ solution with the pH adjusted to 2.8 using phosphoric acid and mobile phase B was MeOH. A two-step linear gradient program was applied: 50:50 (A:B, *v*/*v*) to 25:75 (A:B, *v*/*v*) in the first 14 min, then back to 50:50 (A:B, *v*/*v*) until 20 min. The flow rate was set to 1 mL/min and the chromatograms were registered at a wavelength of 355 ± 4 nm. ChemStation B.04.03 (Agilent Technologies, San Diego, CA, USA) was used for data acquisition and analysis. The retention time of MEL was 14.34 min. The determination coefficient of linearity (*R*^2^) of the (linear) calibration curve was 0.999. The limit of detection (LOD) and limit of quantification (LOQ) values were 0.16 ppm and 0.49 ppm, respectively. EE was calculated using the following equations:

(2) $$\mathrm{EE}\left(\%\right)=\frac{W_{\mathrm{initial}\;\mathrm{drug}}-W_{\mathrm{free}\;\mathrm{drug}}}{W_{\mathrm{initial}\;\mathrm{drug}}}\times 100,$$

where *W*_initial drug_ and *W*_free drug_ were the weight of the total drug and the weight of the free drug, respectively.

### 2.7. Fourier-Transformed Infrared Spectroscopy (FT-IR)

The interaction between MEL and HSA was investigated with FT-IR. The FT-IR spectra of the nanoparticles (in 0.15 g KBr pastille) were recorded on an AVATAR330 FT-IR spectrometer (Thermo Nicolet, Unicam Hungary Ltd., Budapest, Hungary) with a deuterated triglycine sulfate detector. From each sample, 128 scans were performed in the spectral range of 400–4000 cm^−1^ with the spectral resolution of 4 cm^−1^. All spectral evaluations were carried out using OriginPro 8.6 software (OriginLab Corporation, Northampton, MA, USA). The KBr pastilles were pressed with a Specac Hydraulic Press (Specac Inc., Orpington, UK) with 10 tons of pressure.

Changes in the secondary structure of nanoparticles were investigated using the method of least squares. A model spectrum was created from the linear combination of the spectrum of pure MEL and the unique spectra of excipients: x (MEL spectrum) + y (Excipient spectrum) = (Model spectrum) [40]. This model spectrum was compared to the spectra of dried nanoparticle formulations. The broadening and the decreasing of characteristic peaks at different wavenumbers in the measured spectrum compared to the model spectrum can refer to the change in the secondary structure. The x and y coefficients were changed using an iteration method via the Solver extension of Microsoft Excel 2016 (Microsoft, Redmond, WA, USA). It minimized the sum of the squares of the measured spectrum minus the model spectrum differences at each measured wavenumber:

(3) $$\;A_{v}=\;\overset{4000\;{\mathrm{cm}}^{-1}}{\underset{v=400\;{\mathrm{cm}}^{-1}}{\sum }}{\left(A_{v,\mathrm{measured}}\;–\;A_{v,\mathrm{calculated}}\right)}^{2},$$

where *A* is absorbance and *ν* is the wavenumber. Beside the wavenumber of the peak, the unique spectra of the pure components were compared in the product spectra.

### 2.8. Physical Stability

The physical stability of the liquid and freeze-dried solid nanoparticles was investigated according to the circumstances described in the ICH Q1A (R2) guidelines [41]. The Z-average and PdI of the stored samples were determined at 0, 1, 3, and 6 months. Samples during stability testing were stored in a cool place (5 ± 3 °C). Three parallel measurements were carried out at the time points of determination.

### 2.9. In Vitro Dissolution Profiles

The RED Device (Thermo Scientific™, Waltham, MA, USA) was used for the determination of the time-dependent dissolution profiles of MEL API and its formulations (MEL-HSA and MEL-HSA-Tween). A suspension of 2 mg/mL nominal concentration of MEL was prepared in a phosphate buffer (Na_2_HPO_4_–NaH_2_PO_4_, pH 5.6) as a reference for the formulations MEL-HSA and MEL-HSA-Tween. The reference solution was homogenized using an Eppendorf MixMate (Thermo Scientific™, Waltham, MA, USA) vortex mixer for 30 s and an ultrasonic bath (Sonorex Digiplus, Bandelin GmbH & Co. KG, Berlin, Germany) for 10 min. The RED Device inserts (8K MWCO) were fitted into the reusable Teflon base plate, then 100 µL of samples were placed into the donor chambers. Then, 300 µL of phosphate buffer (pH 5.6) was added to the acceptor chambers, and the unit was covered with a sealing tape and incubated at 37 °C on an orbital shaker (at 350 rpm) for 6 h. Samples were taken at different time points from the acceptor chambers and MEL concentrations were determined using HPLC-DAD. For this purpose, three-point calibration curves were generated using a linear regression analysis of the peak areas plotted against known concentrations of MEL in the range of 10–100 µM (5 µL injection volume, *R*^2^ = 1.0000) and 100–1200 µM (1 µL injection volume, *R*^2^ = 0.9993). Each time point per formulation was measured in triplicate.

Quantitative chromatographic analyses were performed on an Agilent 1260 liquid chromatography system equipped with a vacuum degasser, a quaternary pump, a thermostatted autosampler, a column temperature controller, and a diode array detector (Agilent Technologies, Palo Alto, CA, USA). Chromatographic analysis was performed at 40 °C on a Kinetex^®^ 2.6 µm C18 100Å column (30 × 3.0 mm) (Phenomenex, Torrance, CA, USA), with a mobile phase flow rate of 1.0 mL/min. The composition of mobile phase A was 0.1% (*v*/*v*) TFA in water (pH 1.9), mobile phase B was the mixture of MeCN and water 95/5 (*v*/*v*) with 0.1% (*v*/*v*) TFA. A one-step linear gradient program was applied: 2–100% B in the first 3.3 min, then 100% B was kept until 5.50 min, and finally at 5.51 min the percentage of B was dropped to 2%. This was followed by a 1.8 min equilibration period prior to the next injection. Chromatograms were registered at a wavelength of 220 ± 4 nm. ChemStation B.04.03 was used for the data acquisition and analysis. The significance of the differences of dissolution data was calculated with one-way ANOVA with a post hoc test (Tukey’s multiple comparisons test, α = 0.05).

### 2.10. In Vitro Permeability Measurements

A PAMPA system was used to determine the effective permeability of MEL from the suspension of the API and from its HSA-formulations in a comparison study. The same suspension was used as in the case of the dissolution studies as a reference donor solution for the formulations MEL-HSA and MEL-HSA-Tween. The filter donor plate (Multiscreen™-IP, MAIPN4510, pore size 0.45 µm; Millipore, Merck Ltd., Budapest, Hungary) was coated with 5 µL of lipid solution (16 mg PC + 8 mg CHO dissolved in 600 µL dodecane). Then the donor plate was fit into the acceptor plate (Multiscreen Acceptor Plate, MSSACCEPTOR; Millipore, Merck Ltd., Budapest, Hungary) containing 300 μL of PBS solution (pH 7.4), and 150–150 μL of the donor solutions were put on the membrane of the donor plate. The donor plate was covered with a sheet of wet tissue paper and a plate lid was used to avoid evaporation of the solvent. The sandwich system was incubated at 37 °C for 4 h (Heidolph Titramax 1000, Heidolph Instruments, Schwabach, Germany), followed by separation of the PAMPA sandwich plates and the determination of concentrations of MEL in the acceptor solutions using HPLC-DAD. For this purpose, a six-point calibration curve was generated using linear regression analysis of the peak areas plotted against known concentrations of MEL in the range of 1–300 µM (*R*^2^ = 0.9996). For each assay, three replicates per formulations were measured.

The effective permeability and membrane retention of drugs were calculated using the following equation [39]:

(4) $$P_{e}=-\frac{2.303\;\cdot V_{A}}{A\left(t-\tau_{SS}\right)}\cdot \log\left[1-\frac{c_{A}\left(t\right)}{S}\right],$$

where *P_e_* is the effective permeability coefficient (cm/s), *A* is the filter area (0.24 cm^2^), *V_A_* is the volume of the acceptor phase (0.3 cm^3^), *t* is the incubation time (s), *τ_SS_* is the time to reach the steady state (s), *C_A_*(*t*) is the concentration of the compound in the acceptor phase at time point *t* (mol/cm^3^), and *S* is the solubility of MEL in the donor phase. The solubility of MEL in the donor solutions was determined after centrifugation (at 12,000 rpm, 15 mins, Eppendorf Centrifuge 5804 R, Thermo Scientific™, Waltham, MA, USA) in Microcon Centrifugal Filter Devices (30,000 molecular weight cut-off (MWCO)) and 50× dilution of the formulations, using the same HPLC system. The flux of the samples was calculated using the following equation [39]:

(5) $$\mathrm{Flux}=\;P_{e}\cdot S.$$

Quantitative chromatographic analyses were performed using a SHIMADZU Prominence Modular HPLC system equipped with a vacuum degasser, a binary pump, a thermostatted autosampler, a column temperature controller, and a photodiode array detector (Shimadzu Corporation, Kyoto, Japan). Chromatographic analysis was performed at 45 °C on a Cortecs^®^ C18+ 2.7 µm column (50 × 3.0 mm) (Waters Ltd., Budapest, Hungary) with a mobile phase flow rate of 1.0 mL/min. The composition of mobile phase A was 0.1% (*v*/*v*) formic acid in water (pH 1.9), mobile phase B was the mixture of MeCN and water 95/5 (*v*/*v*) with 0.1% (*v*/*v*) formic acid. A one-step linear gradient program was applied: 0% B in the first 0.3 min, then 0–100% between 0.3 and 3.8 min, the 100% B was kept for 1.2 min, and finally at 4.41 min, the percentage of B was dropped to 0%. This was followed by a 1.2 min equilibration period prior to the next injection. Chromatograms were registered at a wavelength of 220 ± 4 nm. LabSolutions v. 5.93 (Shimadzu Corporation, Kyoto, Japan) was used for the data acquisition and analysis.

### 2.11. In Vivo Animal Studies

All experiments involving animal subjects were carried out with the approval of the National Scientific Ethical Committee on Animal Experimentation (permission number: IV/1247/2017). The animals were treated in accordance by the European Communities Council Directives (2010/63/EU) and the Hungarian Act for the Protection of Animals in Research (Article 32 of Act XXVIII). The optimized IN reconstituted formulations contained 2 mg/mL MEL and 10 mg/mL HSA. A 60-µg dose of MEL was administered into the right nostril of 160–180 g male Sprague Dawley rats (*n* = 24) via a pipette. As a control, IV injection prepared using the dilution of a passable injection with a concentration of 15 mg/1.5 mL (Meloxicam-Zentiva, Prague, Czech Republic) containing MEL in solution form (IV MEL) was administered at a dose of 60 µg MEL per rats (*n* = 24). At predetermined time points (5, 15, 30, 60, 120, and 240 min) after the MEL dosing, the blood of the rats—under deep isoflurane anesthesia—was collected into heparinized tubes via cardiac puncture. Then, the animals were sacrificed via decapitation and brain tissues were quickly removed, rinsed in ice-cold PBS, divided into left and right hemispheres, weighed, and stored at −80 °C until assayed.

Plasma samples were centrifuged at 1500× *g* for 10 min at 5 °C. To a 90 µL plasma sample, 10 µL 0.1% aqueous formic acid and 300 µL MeCN containing piroxicam (internal standard at 12.5 ng/mL concentration) were added and the mixture was spun for 60 s. The mixture was allowed to rest for 30 min at −20 °C to support protein precipitation. The supernatant was obtained via the centrifugation of the mixture for 10 min at 10,000× *g* at 4 °C. Twenty microliters of clear supernatant was diluted using 380 µL 0.1% aqueous formic acid and spun for 30 s. Finally, 5 µL was injected into the LC–MS/MS system for analysis. The rat plasma calibration standards of MEL were prepared by moving the working standard solutions (1–1000 ng/mL) into a pool of drug-free rat plasma. The sample preparation procedure described above was followed. The calibration standards containing 6.25 ng/mL and 25 ng/mL MEL were used as quality control (QC) samples and analyzed using LC–MS/MS.

The right hemispheres (0.8–1 g) of rats were homogenized in 5 mL 1% aqueous acetic acid using an Ultra-Turrax^®^ (IKA-Werke GmbH & Co. KG, Staufen in Breisgau, Germany) homogenizer in an ice bath for 2 × 30 s, interrupted by 30 s of cooling. To 100 µL of brain homogenate, 20 µL 0.1% aqueous formic acid and 20 µL piroxicam internal standard (1.56 ng/mL in 0.1% aqueous formic acid) were added and the mixture was vortex-mixed for 60 s. After centrifugation for 10 min at 10,000× *g* at 4 °C, 40 µL supernatant was injected into the LC–MS/MS system for analysis. Rat brain calibration standards of MEL were prepared using the same procedure as in the case of rat plasma calibration.

### 2.12. LC–MS/MS Analysis of In Vivo Studies

The quantitative analysis of MEL was performed after chromatographic separation by using mass spectrometry. An Agilent Liquid Chromatography System series 1100 including a Micro Vacuum Degasser, Capillary Pump, and µ-WPS autosampler (Agilent Technologies, Waldbronn, Germany) connected to a Q ExactiveTM Plus Orbitrap mass spectrometer (Thermo Fisher Scientific, San Jose, CA, USA) equipped with a heated ESI ion source (HESI) was applied to the analysis. Gradient chromatographic separation was performed at room temperature on a Luna C8(2) Mercury column (20 mm × 2.0 mm, particle size 5.0 µm) protected by a C8 guard column (2 × 2 mm) (Phenomenex, Torrance, CA, USA) by using ammonium formate (15 mM, pH = 3) as solvent A and MeCN as solvent B (see Table 2). The calibration curve was shown to be linear over the concentration range of 1–1000 ng/mL.

The mass spectrometer was used in a positive mode with the following parameters of the H-ESI source: spray voltage at 3.5 kV, capillary temperature at 253 °C, aux gas heater temperature at 406 °C, sheath gas flow rate at 46 L/h, aux gas flow rate at 11 L/h, sweep gas flow rate at 2 L/h, and S-lens radio frequency (RF) level at 50.0 (source auto-defaults). Parallel-reaction-monitoring (PRM) mode was used for quantifying by monitoring the following transitions: m/z 352 → 115 and 352 → 141 for MEL and m/z 332 → 95 and 332 → 121 for piroxicam. The normalized collision energies (NCEs) for specific quantification were optimized to maximize the sensitivity. The NCE was 24 for MEL and 29 for piroxicam. A valve placed after the analytical column was programmed to switch the flow onto MS only when analytes of interest elute from the column (brain samples: 0.9–2.1 min; plasma samples: 0.7–2.0 min) to prevent excessive contamination of the ion source and ion optics. Washing procedures of the autosampler before and after injecting samples were programmed in order to avoid the carry-over of analytes. Data acquisition and processing were carried out using Xcalibur and Quan Browser (version 4.0.27.19) software (Thermo Fisher Scientific, San Jose, CA, USA).

### 2.13. Pharmacokinetic Studies

The pharmacokinetic parameters were calculated based on the area under the curve (AUC) of the time (min)–concentration (ng/mL) curves for each animal. Statistical analysis was performed with TIBCO Statistica^®^ 13.4 (Statsoft Hungary, Budapest, Hungary). All reported data are means ± SD. The paired *t*-test was used to determine the statistical significance. Changes were considered statistically significant at *p* < 0.05. The ratio of the AUC value, after IN application in the brain in comparison with the AUC of IV administration (absolute bioavailability for brain—% abs. BA for the brain) was determined according to the following formula:

(6) $$\mathrm{abs}.\mathrm{BA}\;\mathrm{for}\;\mathrm{plasma}\;\left(\%\right)=\frac{AUC_{\mathrm{plasma}\;}IN}{AUC_{\mathrm{plasma}\;}IV}\times 100,$$

(7) $$\mathrm{abs}.\mathrm{BA}\;\mathrm{for}\;\mathrm{brain}\;\left(\%\right)=\frac{AUC_{\mathrm{brain}\;}IN}{AUC_{\mathrm{brain}\;}IV}\times 100.$$

The drug targeting efficiency index (DTE) describes the relative exposure of the brain to the drug following IN administration versus systemic administration was calculated according to the following formula:

(8) $$\mathrm{DTE}\;\left(\%\right)=\frac{\left(\frac{AUC_{\mathrm{brain}\;}}{AUC_{\mathrm{plasma}\;}}\right)IN}{\left(\frac{AUC_{\mathrm{brain}\;}}{AUC_{\mathrm{plasma}\;}}\right)IV}\times 100.$$

The value of DTE can range from −∞ to ∞, and the values higher than 100% indicate more efficient drug delivery to the brain following IN administration when compared to the systemic administration [42,43].

The mean residence time (MRT) is the average time the drug spends in the brain before being eliminated. It was calculated with the following equation:

(9) $$\mathrm{MRT}=\frac{\int C\cdot tdt}{\int Cdt}=\frac{AUMC}{AUC},$$

where AUMC is the first moment of the concentration–time integral, or the AUC formed by time and the product of concentration and time [22].

## 3. Results

### 3.1. Initial Risk Assessment and Knowledge Space Development

The previously defined QTPP was a protein containing a nanosized powder formula for nasal administration, with the proper physical stability of the nanosystem and optimal pharmacokinetic profile for nasal absorption, in order to reach the brain tissue via this alternative route without a first-pass effect, which can be applied by an adult population diagnosed with a mental disorder. The area of potential risks in the formulation development of HSA as a carrier are diverse. These risks can be divided into several groups related to the albumin as a special carrier, which needs special handling during the manufacturing; risks can also be related to the final product (e.g., physical stability, osmotic activity, esterase activity, dissolution, and permeability issues), as well as the therapeutic use (special attention on the application and handling of the HSA containing product regarding its form, i.e., liquid or solid). Further risk groups are the patient (e.g., its immunological reaction) and the regulatory or the authorized body, which may require additional data for proving the safety, efficacy, and quality of the product in the submissions during the authorization process [34].

**Figure 2:**
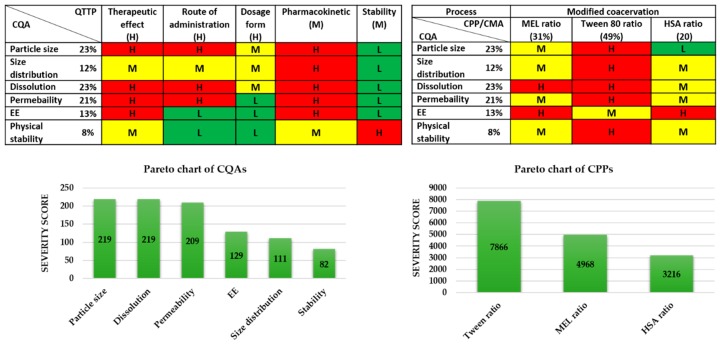
Risk assessment of the MEL-albumin nanoparticle formulation. CMA: Critical material attribute; CPP: Critical process parameter; CQA: Critical quality attribute; EE: Encapsulation efficacy; L,M,H: Low, medium, high.

All of the potential factors, which can influence the targeted protein-containing nasal powder product, were collected and grouped into four sections, as they are related to the material characteristics (like features of the API and excipients), the production process (e.g., the parameters of the lyophilization process), the therapeutic expectations (like target patient population, therapeutic effect, etc.), and the final product characteristics (e.g., nanosized range, nasal route of administration, stability etc.). This collection of the influencing factors is named the “knowledge space development” [30] and helped in the selection of critical factors for performing the initial risk assessment. The selected critical factors are listed below. To the modified coacervation production process, the following are linked as CPPs: the ratio of the active agent (MEL), the ratio of the Tween, and the ratio of the HSA. All the process parameters were kept fixed during the experiments and they were identified according to the previous research results by the research group. Then, the CQAs and the CPPs were also selected. For the CQAs, the following were identified: particle size of the products and the size distribution, the dissolution, the permeability, the EE, and the physical stability of the samples, as these factors have critical effects on the QTPP. Among the QTPP elements, CQAs and CPPs, as well as the CMAs in the initial RA, were performed (Figure 2).

The tables of Figure 2 present the estimated interrelationships of the selected critical factors. Those that have a minimal effect on each other are marked with green, the factors with the medium effect are marked with yellow, and the factors with a great impact are marked with red. As a result, the Pareto charts of the CQAs and CPPs show the theoretical ranking of the critical factors according to their estimated effect on the quality of the targeted final product. It was found that theoretically among the CQAs, the particle size, dissolution, permeability, and EE can be predicted to have the greatest effect on the quality of the targeted and desired nanoparticle (NP) product. The analogous diagram shows that among the CPPs, the ratio of Tween was expected to have the highest influence on the quality of the aimed product. The results of the RA gave the basis of the factorial experimental design. The experimental design pattern was built up based on the ratio of Tween as critical factors of the formulation. As CQAs, particle size and EE can be primarily optimized with the appropriate composition, where these parameters affect the dissolution and permeability.

### 3.2. Optimization of MEL-HSA Formulations via a Box-Behnken Design

Using a Box–Behnken experimental design, the ratio of MEL and HSA, along with the necessity of surfactant Tween 80, were investigated. The experimental conditions and the observed responses for the 15 formulations were analyzed using TIBCO Statistica^®^ 13.4 software and are shown in Table 3.

**Figure 3:**
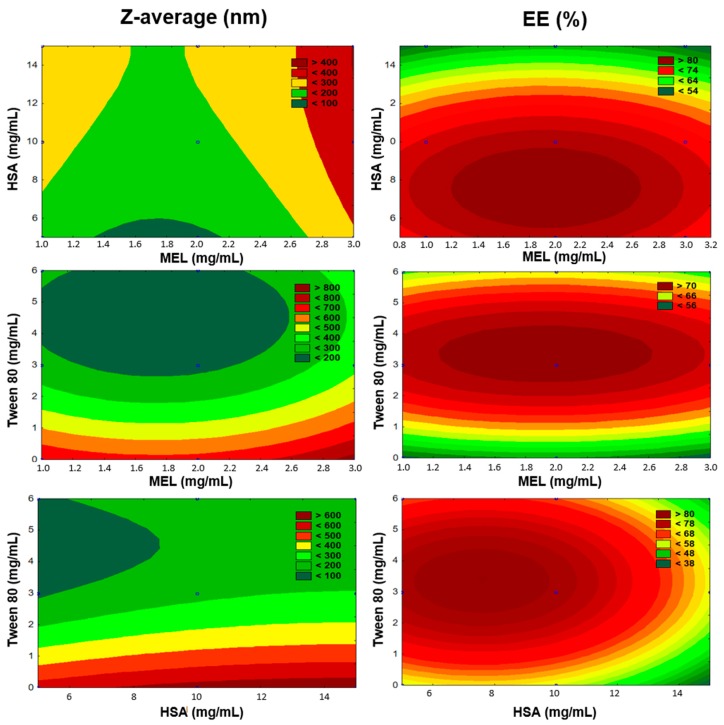
Surface plots of Box-Behnken experimental design.

The number of experiments included the replicated center points. Polynomial equations were generated to describe the individual main effects and interaction effects of independent factors on each dependent factor. The surface plots of the Z-average and EE are shown in Figure 3.

According to multiple regression analysis on the experimental data, the relationship of the variables on Z-average (*Y*_1_) could be described using the following equation:

(10) $$Y_{1}=395.97+67.83x_{1}+59.31x_{2}-236.11x_{3}+10.31x_{1}x_{2}-57.54x_{1}x_{3}-0.57x_{2}x_{3}-65.83x_{1}^{2}+15.33x_{2}^{2}-114.57x_{3}^{2}.$$

The regression coefficient of the surface plot was 0.841 (*R*^2^). We found that the amount of Tween 80 (*x*_3_) was the only factor that significantly favorably affected the Z-average. The positive coefficients before independent variables of the quadratic model indicate an unfavorable effect, while the negative coefficients indicate a favorable effect on the Z-average because our criteria was to formulate nanoparticles with a particle size in the range 100–200 nm (see Table 1). Applying Tween 80 between 3–6 mg/mL in the formulations reduced the particle size. Tween 80 increased the dispersion of the formed nanoparticles in the aqueous medium by coating their surface, therefore avoiding aggregation into bigger particles. In the case of preparing the formulations without Tween, we did not succeed in reaching the desired particle size. The high HSA concentration (>10 mg/mL) showed a negative effect on Z-average. Applying a 2 mg/mL concentration of MEL resulted in optimal product properties.

The effect of the factors on EE (*Y*_2_) could be described with the following equation:

(11) $$Y_{2}=58.64-0.48x_{1}-10.05x_{2}+4.10x_{3}+2.67x_{1}x_{2}+12.89x_{1}x_{3}-2.98x_{2}x_{3}+1.70x_{1}^{2}+5.21x_{2}^{2}-8.68x_{3}^{2}.$$

The regression coefficient of the surface plot was 0.874 (*R*^2^). We found that the amount of HSA (*x*_2_), the concentration of Tween 80 (*x*_3_), and the interactive influence of MEL and Tween 80 (*x*_1_*x*_3_) were the main factors that significantly affected the EE. The positive coefficients before the independent variables of the quadratic model indicated a favorable effect on the EE, while the negative coefficients indicate an unfavorable effect on the EE. Tween 80 had a positive effect on the EE, and the surface plots depicted that the highest EE was reached in the case of using 3 mg/mL. This can be explained with the fact that using surfactants in higher concentrations solubilized MEL and hindered the conjugation to has, which resulted in blank nanoparticles. The largest absolute value of coefficients was the amount of HSA (*x*_2_), indicating that its effect was found to be the main influential factor and had a significant and negative effect on *Y*_2_ in the case of using a higher concentration than 10 mg/mL in the formulation. Applying a 2 mg/mL concentration of MEL showed the expected EE.

### 3.3. Preparation of MEL-HSA Nanoparticles

Based on the Box–Behnken experimental design, a 2 mg/mL MEL concentration showed the desired Z-average and EE values; therefore, the optimized formulation contained 2 mg/mL MEL, 10 mg/mL HSA, and 3mg/mL Tween 80 (which is under critical micelle concentration) (MEL-HSA-Tween). This composition resulted in a 176 ± 0.3 nm Z-average, 0.205 ± 0.01 PdI, −14.1 ± 0.7 mV, and 79 ± 0.5% EE after reconstitution of the freeze-dried sample. As a comparison, the formulation without Tween 80 (MEL-HSA) was also prepared for further investigations.

### 3.4. FT-IR Studies

**Figure 4:**
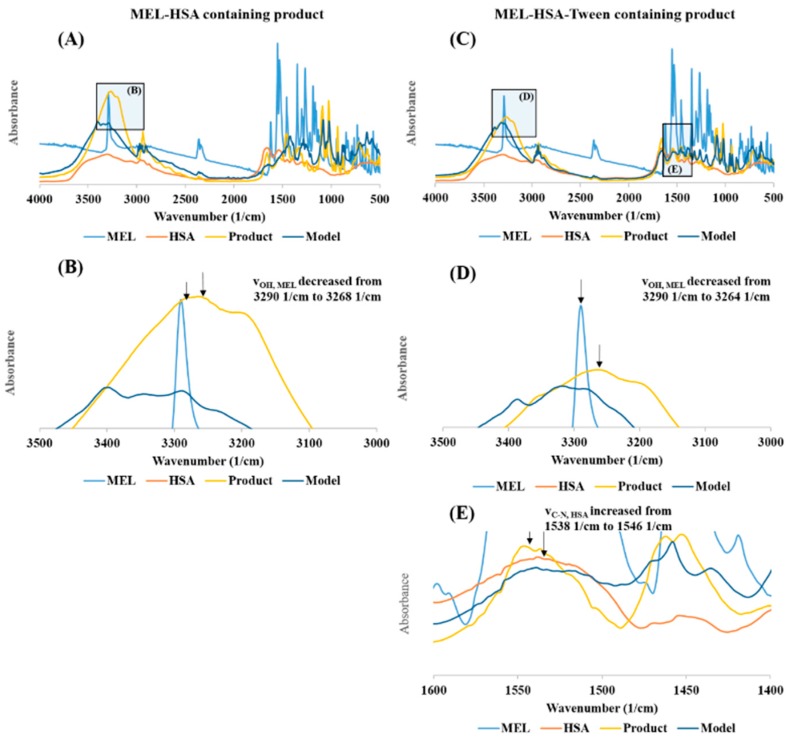
The FT-IR spectra of MEL-HSA (**A**), magnification of MEL-HSA *v*_OH_ region (**B**), MEL-HSA-Tween (**C**), magnification of MEL-HAS-Tween *v*_OH_ region (**D**), and amide II region of HSA (**E**).

The FT-IR investigation showed a secondary chemical interaction between MEL and HSA (Figure 4). The peak maximum of the secondary νOH of MEL also significantly decreased from 3290 cm^−1^ to 3264 cm^−1^ while displaying widening of the broadening; this phenomenon refers to the formation of intermolecular H-bonds, both in MEL-HSA (Figure 4B) and MEL-HSA-Tween (Figure 4D) formulations. Secondary bonds were formed between the MEL and HSA. The measured spectra were decreased compared to model spectra as another sign of the formation of the bonds. Pure HSA had two characteristic vibration bands at 1659 cm^−1^ (amide I) assigned to the stretching vibration of −C=O and 1538 cm^−1^ (amide II) assigned to the C–N stretching vibration and N–H bending vibration investigating with FT-IR. The FT-IR spectra of nanoparticles showed the dominant band of amide I and II, but the position of the amide II peak shifted from 1538 cm^−1^ to 1546 cm^−1^, which suggests the change in the secondary structure of has; therefore, the conjugation with MEL was remarkable in the FT-IR spectra of MEL-HSA-Tween (Figure 4E).

### 3.5. Physical Stability

**Figure 5:**
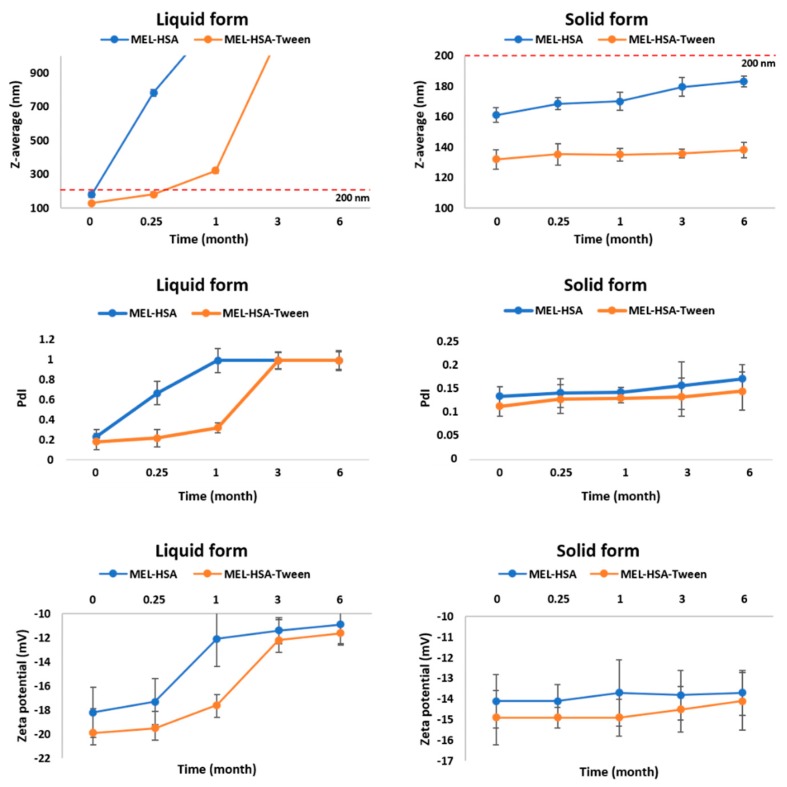
Changes in physical stability during storage.

For physical stability investigations at 5 ± 3 °C, MEL-HSA and MEL-HSA-Tween formulations were prepared both without freeze-drying (liquid form) and with freeze-drying (solid form). The results of the physical stability investigations are presented in Figure 5. The Z-average, PdI, and zeta potential of the liquid and solid forms after reconstitution with purified water were determined at the given time points.

In the case of liquid MEL-has, the Z-average, and therefore the PdI, was largely increased after 1 week, which could be explained by the large surface energy between the nanoparticles. The aggregation of nanoparticles reduced the surface energy, which made the system more stable. MEL-HSA-Tween samples showed a higher physical stability because Tween covered the surface of nanoparticles in the formulation, which could provide sufficient steric repulsion between the particles, therefore, slowing down the aggregation [44]. Aggregation occurred even in liquid MEL-HSA-Tween samples after 1 week because of the free migration of nanoparticles, thus long-term physical stability could not be reached in liquid formulations. The increase of the negative zeta potential also confirmed this fact. In the case of the freeze-dried solid forms, both formulations remained physically stable, their Z-average increased non-significantly and did not reach 200 nm, PdI was under 0.2, and no change was observed in the zeta potential. The tendency of the Z-average increase was lower in the case of the solid MEL-HSA-Tween formulation, which could be explained also with the protective effect of Tween. The PdI and zeta potential of the solid samples were unchanged. Based on these results for further in vitro and in vivo studies, the solid MEL-HSA and MEL-HSA-Tween formulations were reconstituted prior to direct investigation.

### 3.6. In Vitro Dissolution Profiles

**Figure 6:**
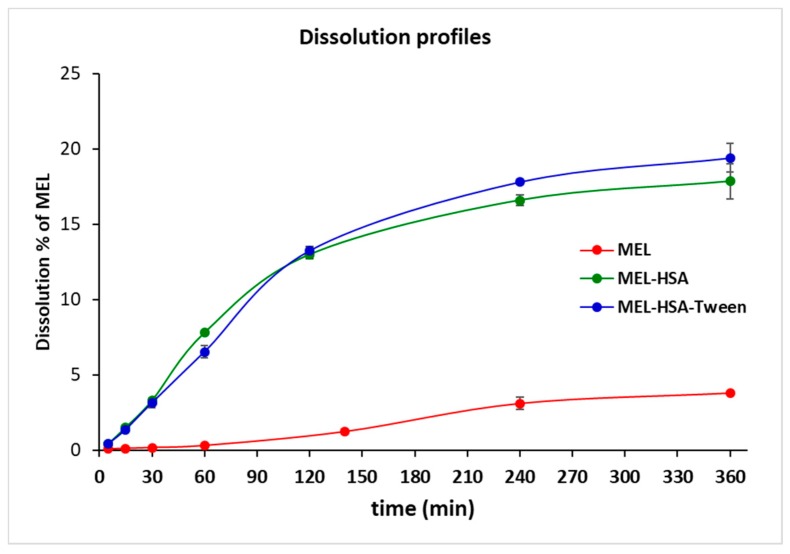
Rapid equilibrium dialysis (RED) of MEL-albumin nanoparticles in comparison with solid MEL.

Plasma protein binding plays an important role in the pharmacokinetics of a drug. Only a free unbound drug can trigger the pharmacological effects on the receptor. There are numerous methods available for measuring plasma protein binding including ultrafiltration, equilibrium dialysis, ultracentrifugation, and in vivo microdialysis. Each technique has its own advantages and disadvantages. Equilibrium dialysis (ED) is the most commonly used method and is frequently considered the gold standard [45]. An RED device was developed in order to reduce the time to equilibrium, and therefore providing results faster than using other methods [37]. RED improves the accuracy of measurements and minimizes inaccuracy due to volumetric deviations in measurements. As the physiological property of HSA is the high protein binding property, RED is a suitable method for the determination of the in vitro drug release of MEL conjugated HSA nanoparticles. The molecular weight cut-off of a semipermeable membrane of RED plate is 8 kDa, which allows only for the free MEL to permeate into the acceptor media. The time-dependent in vitro release profiles of MEL and formulations were determined with RED (Figure 6).

**Figure 7:**
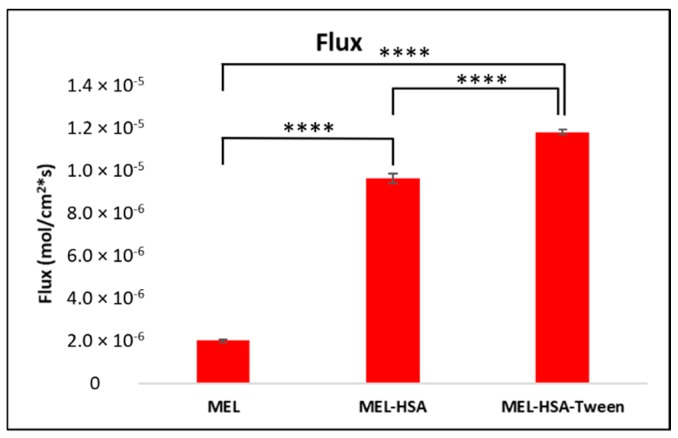
Fluxes of the PAMPA permeability study of MEL-albumin nanoparticles compared to solid MEL. Statistical analysis: Tukey’s multiple comparison test. **** *p* < 0.001 compared to MEL control.

The in vitro dissolution profiles of nanoparticles and solid MEL were investigated in IN circumstances (pH = 5.6). Due to its chemical structure, MEL has a week acidic character (p*K_a_* = 3.43, [39]); therefore, its solubility in this medium was poor (6.12 ± 0.19 µg/mL, at 60 min 37 °C). Both MEL-HSA and MEL-HSA-Tween clearly demonstrated that their nanosize and increased specific surface area resulted in a significantly higher dissolution rate than that of pure MEL (****, *p* < 0.0001). For the MEL-HSA and MEL-HSA-Tween samples, a rapid initial phase was observed for 60 min, followed by a slowing but rising profile. Compared to pure MEL, about a 5 times higher amount, approximately 20% of MEL, was dissolved from the formations within 4 h. Beside this significant increase, we cannot overlook the fact that in the case of the two HSA formulations, the bounded form of MEL was also dissolved due to its rather high binding affinity to HSA (<99%) [46]. At time point of 240 min, the dissolution of MEL from MEL-HSA-Tween was slightly higher than that from the MEL-HSA formulation, but the difference was still significant (**, *p* = 0.0077), which might be explained by the solubilizing effect of Tween as a surfactant additive. This effect of Tween could be also observed in the flux values in the PAMPA study (Figure 7).

### 3.7. In Vitro Permeability Measurements

The PAMPA permeability results of MEL-HSA and MEL-HSA-Tween in comparison to solid MEL are shown in Figure 7. Both nanoparticle formulations showed a significantly higher flux compared to pure MEL (MEL-HSA vs. MEL ****, *p* < 0.001; MEL-HSA-Tween vs. MEL ****, *p* < 0.001). The increased flux of albumin nanoparticles could be explained by the enhanced solubility of MEL due to the solubilizing effect of HSA. When comparing the flux of MEL-HSA to MEL-HSA-Tween, a significant difference was observed (MEL-HSA vs. MEL-HSA-Tween ****, *p* < 0.001); thus, beside HSA, Tween also had a remarkable influence on the flux.

### 3.8. In Vivo Animal Studies

**Figure 8:**
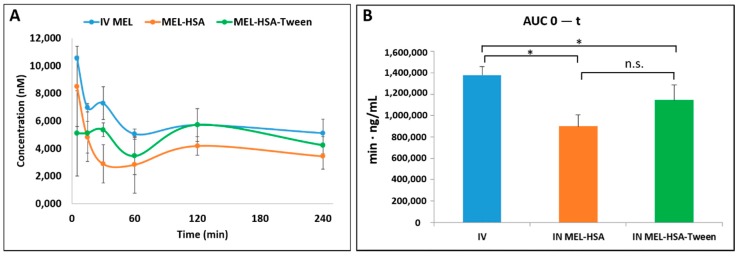
Plasma drug concentration vs. time profiles in rats after intravenous (IV) MEL and intranasal (IN) administration of MEL-albumin nanoparticles (**A**) and their area under the curve (AUC) value (**B**). Statistical analysis: t-test. * *p* < 0.05, n.s. means not significant compared to IV MEL control.

Pharmacokinetic studies were used to determine whether the albumin nanoparticles were able to improve the bioavailability of MEL. Changes in the MEL concentration in the blood plasma as a function of time after the nasal administration of MEL-containing albumin nanoparticles and as a control with the IV injection of MEL are shown in Figure 8A. The plasma concentration of MEL was remarkably higher with the IV administration in the first 60 min. After the IN administration of both nano-albumin formulations, the highest plasma concentrations of MEL were reached after five minutes. There were no significant differences between the plasma concentrations of MEL-HSA and MEL-HSA-Tween. In both cases, a controlled release of drugs was observed one hour after the administration of IN albumin formulations. The AUC of plasma concentration–time was proportional to the amount of drug absorbed during the investigated time interval (Figure 8B).

**Figure 9:**
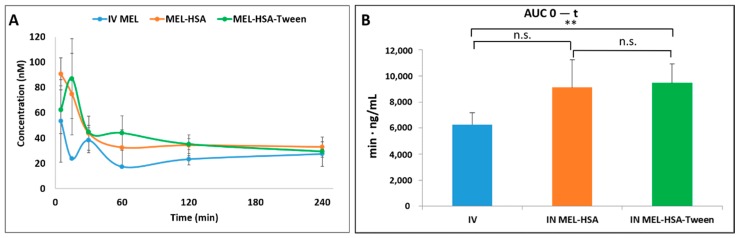
Brain drug concentration versus time profiles in rats after IV MEL and IN administration of MEL-albumin nanoparticles (**A**) and their AUC values (**B**). Statistical analysis: *t*-test. *0 *p* < 0.01, n.s. means not significant compared to IV MEL control.

The plasma AUC values of albumin nanoparticles were similar (MEL-HSA: 899,058 ± 108,514 µmol/mL *, min; MEL-HSA-Tween: 1,146,185 ± 139,954 µmol/mL *, min). The AUC values of both IN administered MEL-HSA and MEL-HSA-Tween was significantly lower (*; *p* < 0.05) than in the case of IV administration (1,377,352 ± 82,059 µmol/mL *, min) (see Figure 8B). This phenomenon could be explained by the 100% bioavailability of drugs after the IV administration, which is always lower in the case of extravascular administration routes. The MEL concentration in the brain versus time profiles in the case of IV and IN administration are shown in Figure 9A. The IN administration of MEL-containing nanoparticles resulted in remarkably higher drug concentration in the brain tissues compared to the IV MEL. The direct “nose-to-brain” transport of the MEL was presumed for both IN albumin formulations because the drug appeared in the brain in the first five minutes after administration. This would not be reached by the absorption from the nasal mucosa to the systemic circulation because of the barrier function of the BBB. After 15 min, the concentration of MEL decreased but held a relatively constant level. This phenomenon could be explained by the residence time of nanoparticles on the nasal mucosa and the adverse effect of mucociliary clearance. Due to the constant MEL brain level after 15 min to 240 min, the endpoint of the investigation can be claimed to be the interaction of the HSA and mucin on the surface of the nasal mucosa. HSA was able to act on mucin (possibly with hydrophobic interactions), forming high-viscosity, gel-like mucin-HSA associates with bioadhesive properties [45]. The calculated cerebral AUC values of the formulations are illustrated in Figure 9B.

Both IN albumin formulations had higher AUC than IV MEL administration. The difference between MEL-HSA—MEL-HSA-Tween (*p* = 0.428) and MEL-HSA—IV MEL (*p* = 0.052) was not significant but the AUC of MEL-HSA-Tween was significantly higher (**, *p* = 0.007) than IV reference. Due to the increased dissolution and permeability of HSA nanoparticles, a high amount of MEL could reach the brain directly by axonal transport, which resulted in higher AUC as compared with IV injection of MEL that might be absorbed through the BBB to target the brain. This result shows that Tween significantly improves the fluidity of the nasal mucosa and in paralell with this the nose-to-brain transport of MEL. The pharmacokinetic parameters of nose-to-brain administration are shown in Table 4.

To determine the utilization of MEL in the brain tissue, the absolute bioavailability was calculated where the cerebral AUC derived from the IV MEL administration was considered 100%. Both HSA formulations reached prominent bioavailability; in the case of nano-MEL-HSA, the absolute bioavailability of MEL was 93.9%, while in the case of MEL-HSA-Tween, it was 96.0%. The cerebral DTE reflected the relative accumulation of the drug in the brain following IN administration compared to systemic (IV) administration. In the case of IN formulations, the percentage DTE data were above 100% in both cases. Therefore, the drug delivery to the brain following IN administration was more efficient when compared to systemic administration. This phenomenon could be explained in terms of both axonal and epithelial routes of drugs compared to an IV administration where the APIs could reach the brain tissues only through the BBB.

## 4. Discussion

HSA, as a versatile, biodegradable nanocarrier, is a promising tool for the nose-to-brain delivery of NSAIDs. HSA nanoparticles have numerous advantages in comparison to other colloidal drug delivery systems. They have no immunogenic effect; therefore, they cannot be identified by macrophages and removed by the reticuloendothelial system (RES) [47]. Moreover, HSA nanoparticles with a size below 200 nm have enhanced permeation and retention (EPR) effect, which helps in passive targeting of the conjugated drug [35].

Traditionally, HSA nanoparticles are prepared using a coacervation method with organic solvents and a cytotoxic crosslinking agent as a stabilizer [48]. In this present work, a modified coacervation method was used, as a green technology, without hazardous excipients, such as glutaraldehide. The stability of the nanoparticles was ensured by the pH-dependent precipitation of HSA. The influencing factors of preparation were examined using the QbD concept, which is a form of risk-and-knowledge-based quality management. The key CQAs were selected (Z-average and EE), which could be primarily optimized with the appropriate composition, and affected the dissolution and permeability. Using a Box–Behnken experimental design, the ratio of components (MEL, HSA, Tween) was optimized for Z-average (100–200 nm) and 79 ± 0.5% EE. The optimized formulation contained 2 mg/mL MEL, 10 mg/mL HSA, and 3 mg/mL Tween 80 (MEL-HSA-Tween).

For a comparison study, liquid and solid forms of the previous composition and the composition without Tween (MEL-HSA) were also prepared to investigate the effect of the surfactant. FT-IR studies proved the formation of nanoparticles due to the change in the secondary structure of HSA and by the formation of stabilizing H-bonds between MEL and HSA. Physical stability investigations showed that the solid formulation resulted in no significant change in the Z-average, PdI, and zeta potential, such that it seemed to be more physically stable. However, the solid form had a high osmotic activity, which could dehydrate the nasal mucosa during administration; therefore, immediately before application, it should be reconstituted with water and administered in liquid form.

In vitro dissolution studies carried out using a RED device showed a significantly increased dissolution rate of MEL from both nanoparticle formulations, especially from MEL-HSA-Tween, which could be claimed with the solubilizing effect of Tween. PAMPA permeability studies showed similar results, where the permeability of MEL from the formulations was significantly higher than of solid MEL, which could be explained by the EPR effect of HSA.

The in vivo results confirmed both the trans-epithelial and axonal transport of MEL-HSA and MEL-HSA-Tween. The plasma AUC values were similar and not significantly lower in the case of the administration of IN forms compared with the MEL-containing IV injection. The axonal transport of MEL was assumed when the drug appeared in the brain tissues in the first five minutes after the application of a nasal spray. In terms of cerebral AUC values, the absolute bioavailability was 93.6% and 96.0% for MEL-HSA and MEL-HSA-Tween, respectively, when compared with the IV injection. This could also be related to the advantageous EPR effect of HSA. The IN administration of nanoparticles resulted in a higher relative accumulation of the drugs (% DTE) in the brain by the axonal nose-to-brain transport when compared with IV administration. This indicates a more efficient MEL delivery to the brain following IN administration when compared to the systemic administration.

## 5. Conclusions

We can conclude that we successfully optimized two formulations of MEL containing HSA nanoparticles for nose-to-brain delivery as a potentially applicable “value-added” product against neuroinflammation. FT-IR studies showed changes in the secondary structure of nanoparticle formulations, which referred to the conjugation of MEL and has; therefore, ensuring the characteristic advantageous physicochemical properties of MEL-containing nanoparticles. From a stability point of view, a solid product (Mel-HSA-Tween) can be recommended for further development since it has met the desired critical parameters (176 ± 0.3 nm Z-average, 0.205 ± 0.01 PdI, −14.1 ± 0.7 mV) after 6 months of storage. However, the solid form had a high osmotic activity, which is unfavorable in the case of application in powder form. The nasal application of the solid product may be accomplished by using a special medical device that allows the powder to be dispersed in the purified water “ex tempore” before administration. Both nanoparticle formulations had an improved in vitro dissolution and permeability, which referred to the increased bioavailability. The in vivo animal studies proved this fact and showed the higher brain targetability in the case of IN administration compared to IV MEL. The pharmacokinetic parameters showed a rapid and longer-acting effect in the brain in the case of IN administration. Based on these results, the IN-administrated, MEL-containing HSA nanoparticles are a promising tool for noninvasive treatment of neuroinflammation.

## Figures and Tables

**Table 1:** The elements of the quality target product profile (QTPP), their targets, and justification.

QTPP Element	Target	Justification
Therapeutic effect, target patient population	Adult population Therapeutic target: reach the Central nervous system (CNS) (brain tissue)	Direct or axonal transport of the active pharmaceutical ingredient (API) from the nose to the brain [35]. No first-pass-effect.
Route of administration	Nasal route	The nasal route offers direct access to the CNS [36] The nasally applied dose of the API can be lower than the orally administered one.
Dosage form and design	Protein containing nanosized lyophilized powder	The nanosize range can improve the absorption time, the dissolution can be also improved via size reduction. Powder form is related to the quality and efficacy, as the solid form can improve product stability.
Pharmacokinetics (dissolution profile and absorption time)	10–15 min	The dissolution and absorption are limited by the periodic nasal liquid renewing and mucociliary clearence, which is usually 10–15 min. It is related to the efficacy of the formulation.
Physical stability	Particle size and size distribution stability is required (100–200 nm)	The lyophilized powder form can help in the protection of the protein and preserve the required particle size. Particle size <100 nm raises nanotoxicity issues. Human serum albumin (HSA) nanoparticles with a size below 200 nm have an enhanced permeation and retention (EPR) effect [37]. It is linked to the quality, safety, and efficacy profile of the preparation.

**Table 2:** The gradient applied for analysis.

Time (min)	Eluent B (%)	Flow Rate (µL/min)
0	40	250
0.5	40	250
2	70	250
2.1	90	600
2.5	90	600
2.6	40	600
4.0	40	600
4.1	40	250
4.5	40	250

**Table 3:** Compositions of the Box–Behnken experimental design and their observed responses.

Standard Run	Independent Variables/Factors			Z-Average (nm)	EE (%)
	MEL (mg/mL)	HSA (mg/mL)	Tween 80 (mg/mL)		
1	1.0	5.0	3.0	371.5 ± 3.8	68.4 ± 1.7
2	3.0	5.0	3.0	168.3 ± 4.1	72.4 ± 3.1
3	1.0	15.0	3.0	282.4 ± 6.2	53.5 ± 2.4
4	3.0	15.0	3.0	285.3 ± 7.5	68.1 ± 1.9
5	1.0	10.0	0	481.3 ± 8.1	71.6 ± 1.2
6	3.0	10.0	0	1198 ± 13.9	34.6 ± 3.5
7	1.0	10.0	6.0	220.6 ± 6.8	56.9 ± 0.9
8	3.0	10.0	6.0	246.8 ± 3.9	71.5 ± 1.4
9	2.0	5.0	0	423.4 ± 5.5	61.3 ± 2.3
10	2.0	15.0	0	663.8 ± 9.1	36.7 ± 3.2
11	2.0	5.0	6.0	102 ± 6	72.6 ± 1.8
12	2.0	15.0	6.0	308.2 ± 7.7	36 ± 3.7
13	2.0	10.0	3.0	175.6 ± 6.8	79.1 ± 2
14	2.0	10.0	3.0	176.1 ± 2.7	80 ± 1.8
15	2.0	10.0	3.0	175.9 ± 4.2	79.2 ± 2.1

**Table 4:** Pharmacokinetic parameters of IV and nose-to-brain administration.

Pharmacokinetic Parameter	Formulation		
	MEL	MEL-HSA	MEL-HSA-Tween
Administration	IV	IN	IN
Ke (min^−1^)	0.00213	0.00272	0.00231
t_1/2_ (h)	6.8 ± 3.1	4.7 ± 1.5	5.5 ± 1.6
AUC 0–t (μmol/mL·min)	626,296 ± 89,313	912,131 ± 212,767	946,683 ± 145,358
AUC 0–∞ (μmol/mL·min)	24,279 ± 12,254	22,788 ± 6139	23,300 ± 2539
Cl (μg/kg)/(μmol/mL)/min	0.0033 ± 0.0016	0.0029 ± 0.0001	0.0026 ± 0.0002
Mean residence time (h)	10.5 ± 4.5	7.3 ± 2.1	8.0 ± 2.2
Drug targeting efficiency (%)	100	223	182
Absolute bioavailability (plasma) (%)	100	65.3	83.2
Absolute bioavailability (brain) (%)	100	93.9	96.0
